# Mental Health Impacts of Wildfire, Flooding and COVID-19 on Fort McMurray School Board Staff and Other Employees: A Comparative Study

**DOI:** 10.3390/ijerph19010435

**Published:** 2021-12-31

**Authors:** Belinda Agyapong, Ejemai Eboreime, Reham Shalaby, Hannah Pazderka, Gloria Obuobi-Donkor, Medard K. Adu, Wanying Mao, Folajinmi Oluwasina, Ernest Owusu, Andrew J. Greenshaw, Vincent I. O. Agyapong

**Affiliations:** 1 Global Psychological e-Health Foundation, Edmonton, AB T6G 2E1, Canada; bagyapong222@gmail.com; 2 Department of Psychiatry, University of Alberta, Edmonton, AB T6G 2E1, Canada; eboreime@ualberta.ca (E.E.); rshalaby@ualberta.ca (R.S.); hannah.pazderka@gmail.com (H.P.); obuobido@ualberta.ca (G.O.-D.); medard@ualberta.ca (M.K.A.); wmao2@ualberta.ca (W.M.); folajinm@ualberta.ca (F.O.); ernestkwame736@gmail.com (E.O.); andy.greenshaw@ualberta.ca (A.J.G.); 3 Vincent Israel Opoku Agyapong Department of Psychiatry, Faculty of Medicine, Dalhousie University, 5909 Veterans Memorial Lane, 8th Floor, Abbie J. Lane Memorial Building, QEII Health Sciences Centre, Halifax, NS B3H 2E2, Canada

**Keywords:** anxiety, depression, post-traumatic stress, floods, COVID-19, wildfire, Fort McMurray

## Abstract

Background: Fort McMurray, a city in northern Alberta, Canada, has experienced multiple traumas in the last five years, including the 2016 wildfire, the 2020 floods, and the COVID-19 pandemic. Eighteen months after the wildfire, major depressive disorder (MDD), generalized anxiety disorder (GAD), and Post Traumatic Stress Disorder (PTSD) symptoms were elevated among school board employees in the city. Objective: This study aimed to compare employees of the school board and other employees of Fort McMurray in respect to the impact the 2016 wildfires, the 2019 COVID pandemic, and the 2020 floods had on their mental health. Methodology: A quantitative cross-sectional survey was conducted in Fort McMurray from 24 April to 2 June 2021. Online questionnaires were administered through REDCap and were designed to capture socio-demographic characteristics, clinical as well as wildfire, COVID-19, and flooding-related variables. Mental health outcome variables were captured using self-reported standardized assessment scales. Data were analysed with descriptive statistics, Chi-square/Fisher’s Exact tests, and binary regression analysis. Results: Of the 249 residents who accessed the online survey, 186 completed the survey, giving a response rate of 74.7%. Of these respondents, 93.5% (174) indicated their employment status and were included in the Chi-square analysis. Most of the respondents were female (86.2%, (150)), above 40 years (53.4%, (93)), and were in a relationship (71.3%, (124)). The prevalence values for MDD, GAD and PTSD among respondents were 42.4%, 41.0, and 36.8%, respectively. There was a statistically significant difference between employees of the school board and other employees with respect to likely PTSD prevalence (28% vs. 45%, respectively, *p* < 0.05), although with other factors controlled for, in a binary logistic regression model, employer type did not significantly predict likely PTSD. Conclusions: The study has established that likely PTSD symptoms were significantly higher in other employees compared to those of school board employees. Greater exposure to the traumatic events and a greater perceived lack of support from other employers might have contributed to the significantly higher prevalence of PTSD in other employees.

## 1. Introduction

Residents of Fort McMurray have suffered a lot of misfortunes in the past five years. The disastrous fire outbreak in 2016 destroyed many homes and businesses and caused the evacuation of everyone in the city. The wildfire burnt 5890 km^2^ of land [1,2] and destroyed 1800 single-family homes, approximately 8% of private dwellings [2,3]. The estimated cost of the wildfire was CAD $10 billion, with CAD $3.7 billion paid out by insurance providers, and the cost is expected to rise until 2026 [3]. Though most of the incurred costs are direct, the indirect costs such as mental health treatment for residents and loss of timber is $1 billion [3]. More than 43% of Canadians experience some major disasters, with 3% exposed to wildfire and 1% to major residential fires [4]. Whether the exposure to disasters is indirect or direct, it can have a major impact on the social and psychological functioning of individuals [5]. The destruction of property, loss of life, plus physical injuries caused by natural disasters in a community, can result in both immediate and perpetual mental health complications including depression, anxiety, post-traumatic stress disorder (PTSD), and substance use disorders [1,6,7,8,9]. Eighteen months after the 2016 Fort McMurray wildfires, the one-month prevalence for likely Major Depressive Disorder (MDD), Generalized Anxiety Disorder (GAD), and PTSD among the school board staff in Fort McMurray were 18.3, 15.7, and 10.2%, respectively [6].

Then, the discovery of the coronavirus disease (COVID-19) in Wuhan, China, and its subsequent declaration as a global pandemic with no definite treatment resulted in profound fear [10,11]. Just like the rest of the world, the city of Fort McMurray was not spared from the havoc of COVID-19. As of Monday, 10 May 2021, there were 1481 active cases of COVID-19 in the city of 66,500 excluding infections detected at work camps outside the city, implying the total number of cases in the region was much higher [12]. The COVID-19 pandemic has been a challenge to everyone’s mental stability [13]. Individuals infected with COVID-19 are at increased risk of developing mental health disorders such as depression, anxiety, and sleep disturbance [14]. The pooled prevalence of stress, depression, and anxiety has been estimated at 29.6%, 33.7%, and 31.9%, respectively, among the general population during the pandemic [15]. Again, the pooled prevalence of depression, anxiety, and PTSD was, respectively, 21.7%, 22.1%, and PTSD 21.5% among healthcare workers [16]. These figures were even higher among COVID-19 patients for depression and anxiety, respectively, at 45% and 47% [14]. During COVID-19, teachers had to abruptly move to distance or online education and had limited access to social support, which functions as a cushion in experiencing stress. A study in Poland found that teachers experienced at least mild levels of stress, anxiety, and depression, both during both the first and second waves of the COVID-19 pandemic [17].

In an effort to reduce the spread of the COVID-19 virus, governments worldwide introduced restrictive measures, resulting in social isolation, which unfortunately impacts mental health [18]. Though quarantine and self-isolation have been beneficial in combating COVID-19, they frequently result in adverse psychological effects such as anxiety, depression, and post-traumatic stress disorder (PTSD) [19]. Consequently, the public, including patients and health professionals, has been under enormous psychological stress [20]. A cross-sectional study conducted in Spain to assess mental health consequences during COVID-19 indicated that 18.7% of the sample had depressive symptoms, 21.6% anxiety and 15.8% PTSD symptoms [21]. In another study of Text4Hope subscribers in the Province of Alberta, Canada, the one-week prevalence for self-reported measures of moderate to high stress, likely Generalized Anxiety Disorder (GAD), and likely Major Depressive Disorder (MDD) were 84.9%, 46.7%, and 41.4%, respectively [22]. The 6-week prevalence rates of moderate or high stress, anxiety, and depression symptoms were 85.6%, 47.0%, and 44.0%, respectively [23]. In the midst of the escalating pandemic in the region, in May of 2020, Fort McMurray was further hit with severe flooding. This phenomenon is one of the most common and destructive natural disasters worldwide [24,25], typically causing bodily harm and mental health challenges like PTSD, affecting individuals’ social and functional capabilities [24]. Flooding disasters may have negative effects on the mental health of survivors for a prolonged period [24]. After such disasters, social and psychological factors may play a huge role in speeding or hindering recovery among affected populations [26]. Flooding results in the loss of work, physical possessions, displacement of thousands of residents temporarily if not permanently, and survivors are likely to develop some form of instability due to the stressors of the disaster, leading to compromised resilience [27,28,29]. Flooding can also cause bodily harm and mental health challenges like PTSD, which affects individuals’ social and functional capabilities [24,25]. The effects of flooding on common mental disorders were assessed in high and middle-income countries, and results revealed a significant increase in depression, anxiety, and psychological distress among adults [27,30]. Survivors may also experience psychological stress, physical health ailments, water health-and-safety problems related to the floods, as well as increased psychological health problems [31]. In addition, research by Greene et al. concluded that, in the context of flooding, resilience was strongly associated with lower psychological distress, but there was a correlation between flooding and subsequent poor mental health [29]. Unfortunately, the flooding in Fort McMurray primarily hit the downtown region where most of the school board buildings are located, perhaps putting school board staff at greater risk of mental health ill effects.

These multiple traumas—wildfire, floods, and COVID-19—are predicted to lead to increased anxiety, depression, and other mental health conditions in Fort McMurray. Exposure to natural disasters has also been associated with a small to moderate increase in the risk for common mental health problems [32]. In retrospect, the majority of survivors cope reasonably well with disasters, yet a considerable number of people experience psychological damage, with some at a significantly increased risk of developing mental disorders [27,30]. According to the World Health Organization, the overall prevalence of mental health problems after disasters is expected to double from 10 to 20% for mild-to-moderate difficulties and rise from 2–3% to 3–4% for severe mental disorders [33]. Besides PTSD, depression is the second most frequently reported and assessed mental health problem after a disaster exposure [34]. Notably, PTSD may also contribute to the development of many other disorders including anxiety disorders, major depressive disorder, and mania [35].

Many factors including socio-demographic and clinical variables may predict the likelihood of individuals suffering long-term mental health impacts of disasters. These include pre-existing mental health issues, individual resilience, the severity of the disaster, the degree of involvement, social support and relationships, and support from government [1,24,36,37]. In a previous study in the general Fort McMurray population 6 months after the wildfire, four independent factors were associated with the development of likely MDD: support from family/friends, age, witnessed burning of homes, and history of depressive disorder. However, only the independent factor of support from family/friends predicted likely MDD [38]. Predictors of GAD symptomatology six months after the wildfire disaster were: pre-existing anxiety disorder, witnessing of homes being destroyed by the wildfire, living in a different home after the wildfire, receiving limited governmental support or limited family support, and receiving counseling after the wildfire [27]. The prevalence and correlates of anxiety, depression, and PTSD among school board staff were measured 18 months after the Fort McMurray wildfires [6]. That study did not compare the results to those of non-school board employee residents of the city. School board staff, especially teachers, had to deal with their personal and family stressors arising from multiple disasters while helping to support students under their care. Many of these students exhibited increased anxiety, depression, and PTSD symptoms from the 2016 wildfires and were already more challenging to manage and support. That situation was further compounded by COVID-19 and flooding, increasing the likely mental strain on school board staff. Consequently, it is of interest if school board staff experienced mental health impacts from the multiple disasters which are in any way different from other residents of the city. In view of this, the objective of this study was to compare employees of the school board to other employees of Fort McMurray in respect to the impact of the 2016 wildfires, the COVID-19 pandemic, and the 2020 floods had on their mental health. To our knowledge, this is the first study that examines these issues in school board staff relative to other employees. Specifically, we aim to explore the prevalence of likely depression, anxiety, and PTSD among school board workers compared to other employees, and also examine the differences in associated demographic, clinical, and other trauma-related risk factors.

## 2. Materials and Methods

### 2.1. Study Design

A quantitative cross-sectional survey was conducted in Fort McMurray from 24 April to 2 June 2021. We collected data using online administered questionnaires through REDCap (https://redcap.vanderbilt.edu accessed on 20 April 2021). The questionnaires were designed to collect demographic and clinical as well as wildfire, COVID-19, and flooding related variables. The online survey was distributed via email to residents using government, school, occupational and community platforms.

### 2.2. Ethics Statement and Consent

The protocol was approved by the University of Alberta Review and Ethics Board (Pro00066054). Informed consent was implied by all study participants after they had read the online information leaflet and completed the survey questionnaires in accordance with the Declaration of Helsinki. The online survey included an information leaflet that potential study participants had to access before beginning the survey. The information leaflet advised participants that consent was implied if they completed and submitted the online survey.

### 2.3. Study Setting

Fort McMurray is a city in the Regional Municipality of Wood Buffalo in Northern Alberta. The population of Fort McMurray according to the 2016 census was 66,573 [39]. There are two school districts: the public-school district, which is home to 16 schools, and the Catholic school district with 13 schools. An estimated total of 1446 staff, including 725 (teachers and teaching assistants) and 721 management and support staff was employed in the two school districts in Fort McMurray in November 2018 [6]. The biggest employers in relation to other employees in Fort McMurray are the oil sands mining companies, including Suncor and Syncrude. There is also one main college, Keyano College, the police (RCMP), retail and services industries, and the health sector, among others.

### 2.4. Data Collection Tool and Outcome Measures

Overall, fifty demographic, clinical, wildfire, flooding and pandemic exposure and support variables were collected through an online survey (Supplementary Materials).

Clinical outcomes were assessed using validated screening scales for self-reported symptoms, including the Generalized Anxiety Disorder 7-item (GAD-7) Scale (GAD-7 score ≥10 indicates likely generalized anxiety disorder [GAD]) [40]. The Patient Health Questionnaire-9 (PHQ-9; a score ≥10 indicates likely major depressive disorder (MDD)) [41], and PTSD Checklist Civilian (PCL-C) was used to assess likely PTSD (PCL-C score of 44 or more for likely PTSD) [42] and the Brief Resilience Scale (BRS) (a mean score ≤2.99 on the BRS indicates low resilience) [43]. Validated scales were chosen to better understand support seekers’ self-reported symptoms, potential symptom severity, and to screen for symptomatology. These scales were not intended as diagnostic tools.

### 2.5. Statistical Analysis

Results were analyzed using SPSS Version 25 [44]. Descriptive statistics were provided for demographic, clinical, wildfire, flooding and COVID-19 related variables based on employer type analysis. Cross-tabular analyses using the Chi-square test explored differences between school board employees and other employees with respect to demographic and clinical, and wildfire, flooding, and COVID-19-related variables. Descriptive characteristics were presented as numbers and percentages, and a two-tailed *p* ≤ 0.05 was used to determine statistical significance for all analyses. For any mental health outcome (likely GAD, MDD, PTSD, and low resilience) which showed a significant association with employer type, we planned to use binary logistic regression analysis to assess if employer type was a significant predictor of the mental health outcome, with other relevant variables controlled for.

## 3. Results

Overall, 249 residents accessed the online survey, of which, 186 completed the survey, giving a response rate of 74.7%. Of the respondents, 174 indicated their employment status and were included in the Chi-square analysis. Of the employed, 50% (87) indicated they were employed by the school board and the other 50% had other employers. Of those who had other employers, 10 (4%) were employed in healthcare, 20 (8%) were employed by Keyano College, 13 (5.2%) were employed by oil sands companies, 13 (5.2%) were employed by municipal or government agencies and 31 (12.4%) had other employers. Most of the respondents were female (86.2% (150)), above 40 years of age (53.4% (93)), and were in a relationship (71.3% (124)).

Table 1 indicates significant differences between respondents who were employed by the school board and other employees in respect of three wildfire-related variables. Respondents who were employed by the school boards were significantly less likely to reside in Fort McMurray during the 2016 wildfire, or live-in neighborhoods with 50.1–300.0 properties destroyed per km^2^ or had their homes destroyed by the wildfires. There was no significant difference between the school board employees and other employees with respect to biological sex, age, marital status, or any of the wildfire-related variables.

Table 2 indicates that, compared to the school board employees, other employees differed with respect to their specific areas of residence during the 2020 Fort McMurray flooding as well as the level of support received from family and friends during the flooding. Respondents who were employed by the school board were less likely to reside in flooded areas. In addition, post hoc analysis using adjusted residuals indicates that school board respondents were less likely to report receiving limited support from family and friends during the flooding, compared to other employment (*z* = 2.9, adjusted *p* = 0.03). Other flooding related variables, including damage to home or loss of property due to the flooding, did not differ significantly between school board employees and the others.

Table 3 indicates that the respondents differed significantly in relation to only one variable: “support from employers since the pandemic”. A lower proportion of school board employees selected “not at all” with respect to the level of support received from employers since the pandemic. Variables such as fear of contracting the coronavirus and exposure to coronavirus related news did not differ significantly between the respondents.

Table 4 indicates that there were statistically significant differences between school board employees and others with respect to three clinical variables. Significantly fewer school board employees had received a mental health diagnosis, had been diagnosed specifically with depression, or met the criteria for likely PTSD at the time of data collection. There were no statistically significant differences between school board employees and others with respect to other clinical variables including the diagnosis of an anxiety disorder, receipt of counselling in the past year, receptivity to counselling, currently use of antidepressants or sleeping tablets, or meeting the criteria for MDD or GAD at the time of data collection.

To access if employer type independently predicted likely PTSD when other variables are controlled for, we initially ran a Chi-square analysis to identify demographic, clinical, wildfire, flooding and pandemic variables that had a significant or near significant association with likely PTSD. We then performed a binary logistic regression analysis to assess if employer type was a significant predictor of PTSD with the other variables controlled for. Variables that showed either significance (*p* ≤ 0.05) or near significance (0.1 ≥ *p* > 0.05) obtained from the univariate Chi-squared analysis were included in the regression model. Correlational analysis was performed prior to the regression analyses to exclude any strong intercorrelations (Spearman’s correlation coefficient of 0.7 to 1.0 or −0.7 to −1.0) among predictor variables.

The initial Chi-square analysis revealed significant or near significant association of eighteen variables with likely PTSD, including past depression diagnosis, other mental health diagnosis, currently taking antidepressants, taking sleeping tablets, desire to receive mental health counselling, received mental health counselling, received sufficient support from family and friends after wildfire evacuation, fearful for your life or the lives of your friends or family during the evacuation, received sufficient support from the Red Cross after the wildfire evacuation, area of residence during the 2020 flood, received sufficient support from family and friends after the floods, lost property as a result of the floods, losing job due to the COVID-19 pandemic, received sufficient support from family and friends since the COVID-19 pandemic declared, received sufficient support from employer since the COVID-19 pandemic, received sufficient support from the Government of Alberta since the COVID-19 pandemic, lost job due to the COVID-19 pandemic, and fearful about close friends or family members contracting COVID-19.

Two variables were thus excluded (having received a mental health diagnosis and currently taking antidepressants) from the regression model because they were highly correlated with two other variables. The regression model predicting the likelihood of PTSD symptoms included 18 demographic, wildfire, flooding, and COVID-19 variables. The model was significant, X^2^ (df = 30, *n* = 186) = 116.96, *p* < 0.001, suggesting the model significantly distinguished between respondents who had likely PTSD and those who did not. The model explained between 56.9% (Cox and Snell R2) and 77.2% (Nagelkerke R2) of the variance and correctly classified 87.8% of all cases. Controlling for all demographic characteristics, employer type was not a significant predictor (*p* = 0.97) of likely PTSD.

## 4. Discussion

This study is a component of a larger on-going research project to assess the impact of the Fort McMurray wildfire of 2016, the ongoing COVID-19 pandemic, and the 2020 flood on the mental health of school staff as well as employees of other organizations. Findings from the present study indicate a relatively increased prevalence of PTSD but not MDD and GAD in employees of other organizations compared to school board staff, although with other factors controlled for in a logistic regression model, employer type did not significantly predict likely PTSD. There were also no statistically significant differences between school board employees and others with respect to other clinical variables, including those who had received counselling in the past year, those willing to receive counselling or those who were currently taking antidepressants or sleeping tablets. In addition, there was no difference in resilience levels for the two employee groups.

### 4.1. Post-Traumatic Stress Disorder (PTSD)

Eighteen months after the 2016 Fort McMurray wildfire, PTSD symptoms increased from 0.0% (no individual reported this prior to the wildfire) in employees of the school boards to 10.2% (11.3% in females and 3.4% in males) [6]. There has been a substantial increase in the overall prevalence of likely PTSD (36.8%) five years after the wildfire in a representative sample of the same population, which may be attributed to additional trauma from the subsequent flood as well as the ongoing pandemic. PTSD prevalence in our study falls within the range reported for natural disaster survivors in the literature as well as our prior results including for first responders such as firefighters [27,45], although there are significant differences such as time from the traumatic disaster exposure, exposure to multiple traumas, as well as sample characteristics. For school board staff specifically, there was an increase in the prevalence of PTSD reported, from 10.2% eighteen months after the wildfires [6] to 28.0% five years after the wildfire, as reported in this study. Despite this rise, the prevalence of PTSD among school board employees remained significantly less than the prevalence recorded for other employees (45.0%) five years after the wildfire. Other employees include a broad range of employment which could potentially explain the comparatively increased likelihood of PTSD. For firefighters, armed forces, and workers with no prior training following a disaster, other findings [35] indicate a lower prevalence of PTSD (21%) than that reported for both school board and other employees in our study.

Post-traumatic stress reactions have both immediate and long-term impacts on individuals after trauma exposure, with symptoms usually appearing in the first month after the traumatic event, although in a few cases there can be months or even years of delay before symptom onset [46]. Previous findings suggest females may be more prone to post-disaster PTSD than their male counterparts [47,48]. In this study, most of the respondents were females (86.2%) which may explain a high prevalence of PTSD in the overall sample. There was no significant sex difference between school board employees and other employees, which suggests that sex did not account for a lower PTSD prevalence among school board employees. Similarly, age and marital status did not differ significantly between the two groups and could not have contributed to the difference in likely PTSD observed in the two employee groups.

Respondents’ residence and trauma exposure seem to be influential factors in the prevalence of PTSD. People who live in areas with the worst destruction following natural disasters present with significantly more PTSD symptoms [49]. Respondents in this study who were employed by the school boards were significantly less likely to reside in neighborhoods with 50.1–300.0 properties destroyed by the 2016 wildfires per km^2^ or had their homes destroyed by the wildfires. They were also less likely to reside in flooded areas during the 2020 floods. Thus, school board employees appeared to have had less intense exposure to trauma from the wildfires and the floods. Consequently, they were more protected from subsequent development of PTSD. Published reports indicate that, during the periods of floods, people who lived in affected areas were at much higher risk of developing PTSD symptoms than those who resided in proximal non-affected areas [50,51].

In a comparable study after an earthquake, the degree of exposure, in terms of the distance from the seismic epicenter, was deemed a major risk factor for PTSD, with greater symptom levels reported for individuals who were closer to the epicenter at the time of the earthquake [49,52]. Floods have a negative effect on survivors’ mental health for a prolonged period [24]. The likely re-exposure to another disaster, i.e., the floods with greater severity may have resulted in the increased likely PTSD symptoms in the other employees. On the contrary, the other trauma factors related to the wildfire, flooding, and COVID-19, such as the fear for one’s life and the lives of family and friends during the wildfire and flooding as well as fear of contracting COVID-19 did not differ significantly between the two groups and so might not have influenced the differential prevalence of PTSD between school board employees and others. Similarly, the degree of exposure to trauma-related news items during the wildfires, flooding, and COVID-19 pandemic did not differ significantly between the two groups and so, as mentioned previously, was not a determinant of PTSD prevalence differences between school board employees and others.

### 4.2. Generalized Anxiety Disorders (GAD)

There were no significant differences between school board employees and others in relation to the likelihood for GAD (33.3% vs. 46.3%, respectively, *p* > 0.05). Our study indicates an increased prevalence (33.3% compared to 15.7%) for likely GAD among the school board staff 18 months after the Fort McMurray wildfire [6]. Exposure to natural disasters is associated with a small to moderate increase in risk for common mental health problems including anxiety [32]. This can explain why after 5 years of wildfire and further exposure to floods and the COVID-19 pandemic the estimated risk for likely GAD doubled.

### 4.3. Major Depressive Disorder (MDD)

Significantly less school board employees compared to other employees reported having received any mental health diagnosis (43.7% vs. 60.9%, respectively) or having been diagnosed specifically with depression (21.8% vs. 40.2%, respectively). Notwithstanding, the prevalence of likely MDD (measured by PHQ-9) and the use of antidepressant treatment were not statistically different between school board employees and other employees (35.5% vs. 48.8%, respectively). Such a discrepancy may need further probing with large-scale studies to demonstrate the relationship between the self-reported symptoms and the predefined clinical diagnoses. Other published results suggest, for wildfire disasters, low- to highly-affected communities showed MDD rates of 6.3 and 12.9%, respectively [53]. The one-month prevalence rate for likely MDD eighteen months after the wildfire in school staff in Fort McMurray was 18.3% [6]. The potential explanations for the apparent increase of likely MDD among the school board staff in our study may be due to the ongoing COVID-19 pandemic and the added trauma of the 2020 floods. Meta-analysis has also shown a tremendously wide range of MDD rates, after exposure to natural disasters, with reports between 6 and 54% [54]. Our finding is consistent with this, although sample size, time from the traumatic disaster exposure, exposure to multiple traumas, and data collection techniques are different.

### 4.4. Support/Resiliency

There were significant differences between school board employees and other employees in the perceived level of support received from family and friends during and after the flooding but not during the wildfires or during the COVID-19 pandemic [6]. Among the school board employees, school board respondents were less likely to report receiving limited support from family and friends during the flooding, compared to other employees. Perceived low levels of support from family and friends had an impact on the likely diagnosis of MDD and GAD six months after the Fort McMurray wildfires. Support from family and friends is most frequently utilized in comparison to support from larger governmental or non-governmental organizations during disasters [4]. Similarly, respondents differed significantly in relation to the support from employers since the pandemic. A lower proportion of school board employees selected “not at all” in respect to the level of support received from employers since the pandemic, which can partly explain the lower likely PTSD prevalence reported. The global economic uncertainty due to COVID-19 has led to millions of people losing their jobs or access to employment. Individuals who unfortunately lost their jobs now also face the stress of looking for jobs in the future [55]. The pandemic directly threatened work with an increased number of people losing their jobs. Hence, support from employers is crucial for the mental well-being of people. After a disaster, social and psychological factors may play a huge role in speeding or hindering recovery among affected populations [26], thus the lack of support might have contributed to the increase in the prevalence of PTSD. Overall, as high as 35.0% of respondents had low resilience which is consistent with the literature which suggests that people could be less resilient when exposed to multiple hazards [56]. There was no significant difference between the two employee groups in respect of their level of resilience.

### 4.5. Limitations

This study has several limitations. First, the online survey questions related to wildfire, flooding, and the pandemic were not validated and so it is possible they did not accurately measure what they were intended to measure. Second, the scales used to assess mental health variables, although they were standardized scales, are not meant to be diagnostic. Third, the demographic variables in the study did not reflect the demographics of Fort McMurray and, therefore, the study findings may not be generalizable. For example, there was a predominance of females in our study sample, with less than 14% male participants. Fourth, our study sample was heterogeneous, for example, school board employees including teachers, administrative, and support staff of the public and Catholic school boards. On the other hand, other employees included healthcare workers, oil sands workers, municipal employees, private sector employees and employees of Keyano College, not-for-profit organizations, and those who were self-employed. Fifth, given that the survey was shared via e-mail by community partners, it was not possible to specify how many individuals received the survey links. The study response rate was calculated using the number of unique individuals who clicked on the link as the denominator, rather than the number who received the link, as the latter could not be determined. The method used for response rate estimation assumes that everyone who receives the survey link clicked on the link and could lead to an over estimation of the response rate. These limitations notwithstanding, our study is the first to examine and compare mental health variables in employees of school boards with those of other employees in a city hit by multiple traumas and our findings would be of interest to policy makers, especially those working in the education sector.

## 5. Conclusions

This study compared the mental health impact of wildfire, flooding, and COVID-19 in and between Fort McMurray school board staff and other employees. The study has established that likely PTSD symptoms were significantly higher in other employees compared to those of school board staff, although with other factors controlled for in a logistic regression model, employer type did not significantly predict likely PTSD. There were no significant differences between school board employees and others in respect of other clinical variables including likely MDD and GAD. This study has also identified potential factors contributing to the increased prevalence of likely PTSD symptoms in other employees compared to school board employees. These factors may be useful for policy makers when formulating population-level social and clinical programs, to alleviate the mental health effects of future natural disasters. Further studies are needed to explore and compare the impact of accessible and rapidly implementable mental health interventions such as supportive text messages on school board staff and other employees. Interventions such as supportive and informative text messaging can be used to alleviate the long-term mental health effects of the wildfires, floods, and the COVID-19 pandemic [57,58,59,60,61,62,63,64]. Future studies may also examine the contribution of adverse childhood experiences and personal traumas such as bereavement, domestic violence, and sexual trauma on mental health outcomes in a community affected by multiple natural disasters.

## Figures and Tables

**Table 1:** Chi-square test of association between employer type, the demographic, and the 2016 wildfire related variables.

Variables	Employer		*p*-Value	Chi- Square	Effect Size Phi/Cramer’s V	Overall
	School Board	Other Employment				
**Gender**						
Male	8 (33.3%)	16 (66.7%)	0.123	3.093	0.133	24 (100.0%)
Female	79 (52.7%)	71 (47.3%)				150 (100.0%)
**Age in years**						
≤25	3 (3.4%)	5 (5.7%)	0.710	0.634	0.060	8 (4.6%)
26–40	38 (43.7%)	35 (40.2%)				73 (42.0%)
>40	46 (52.9%)	47 (54.0%)				93 (53.4%)
**Marital Status**						
Married/Partner/Cohabiting	65 (74.7%)	59 (67.8%)	0.339	2.29	0.115	124 (71.3%)
Divorce/Widowed/Separated	6 (6.9%)	12 (13.8%)				18 (10.3%)
Single	16 (18.4%)	16 (18.4%)				32 (18.4%)
**Residence during 2016 wildfires**						
Fort McMurray	71 (84.5%)	78 (95.1%)	0.038	5.07	0.175	149 (89.8%)
Other	13 (15.5%)	4 (4.9%)				17 (10.2%)
**Specific Residence during 2016 wildfire**						
0–1.0 properties destroyed per km^2^	43 (57.3%)	27 (33.8%)	0.002	12.405	0.283	70 (45.2%)
1.1–50.0 properties destroyed per km^2^	21 (28.0%)	23 (28.7%)				44 (28.4%)
50.1–300.0 properties destroyed per km^2^	11 (14.7%)	30 (37.5%)				41 (26.5%)
**Witnessed burning of homes during 2016 wildfires**						
No	16 (19%)	12 (14.6%)	0.536	0.576	0.059	28 (16.9%)
Yes	68 (81.0%)	70 (85.4%)				138 (83.1%)
**Home completely destroyed**						
No	80 (92.0%)	67 (77.0%)	0.011	7.409	0.206	147 (84.5%)
Yes	7 (8.0%)	20 (23.0%)				27 (15.5%)
**Fearful for life/family/friends on evacuation day**						
No	11 (13.1%)	8 (9.8%)	0.627	0.456	0.052	19 (11.4%)
Yes	73 (86.9%)	74 (90.2%)				147 (88.6%)
**Frequency of watching television images related to wildfire devastation during period of evacuation**						
Daily	66 (78.6%)	67 (81.7%)	0.916	0.364	0.047	133 (80.1%)
<Daily	11 (13.1%)	10 (12.2%)				21 (12.7%)
I did not watch the TV images of the devastation	7 (8.3%)	5 (6.1%)				12 (7.2%)
**Frequency of reading newspaper/internet article related to devastation by wildfire during period of evacuation**						
Daily	68 (81.0%)	73 (89.0%)	0.305	2.733	0.128	141 (84.9%)
<Daily	13 (15.5%)	6 (7.3%)				19 (11.4%)
I did not read newspaper and internet articles related to the devastation	3 (3.6%)	3 (3.7%)				6 (3.6%)
**Support from family/friend after the evacuation order**						
Yes. I have had absolute support	59 (72.0%)	52 (63.4%)				111 (67.7%)
Yes. I have had some support	14 (17.1%)	17 (20.7%)	0.141	5.501	0.183	31 (18.9%)
Yes. But only limited support	3 (3.7%)	10 (12.2%)				13 (7.9%)
Not at all	6 (7.3%)	3 (3.7%)				9 (5.5%)
**Support from Red Cross after evacuation order**						
Yes. I have had absolute support	43 (52.4%)	30 (36.6%)				73 (44.5%)
Yes. I have had some support	23 (28.0%)	28 (34.1%)				51 (31.1%)
Yes. But only limited support	7 (8.5%)	11 (13.4%)	0.224	4.421	0.164	18 (11.0%)
Not at all	9 (11.0%)	13 (15.9%)				22 (13.4%)
**Support from Government of Alberta after evacuation order**						
Yes. I have had absolute support	32 (39.5%)	24 (29.3%)				56 (34.4%)
Yes. I have had some support	21 (25.9%)	26 (31.7%)				47 (28.8%)
Yes. But only limited support	12 (14.8%)	17 (20.7%)	0.469	2.563	0.125	29 (17.8%)
Not at all	16 (19.8%)	15 (18.3%)				31 (19.0%)

**Table 2:** Chi-square test of association between employer type and flooding related variables.

Variables	Employer		*p*-Value	Chi- Square	Effect Size Phi/Cramer’s V	Overall
	School Board	Other Employment				
**Specific Residence during 2020 flooding**						
Resided in areas not affected by the floods	75 (90.4%)	63 (75.9%)	0.021	6.186	0.193	138 (83.1%)
Resided in flooded areas	8 (9.6%)	20 (24.1%)				28 (16.9%)
**Witness flooding of homes or structures in Fort McMurray**						
No	22 (27.2%)	17 (20.7%)	0.363	0.925	0.075	39 (23.9%)
Yes	59 (72.8%)	65 (79.3%)				124 (76.1%)
**Fearful for self/family/friends’ life during flooding**						
No	61 (75.3%)	55 (67.1%)	0.300	1.347	0.091	116 (71.2%)
Yes	20 (24.7%)	27 (32.9%)				47 (28.8%)
**Frequency of watching television images related to 2020 flooding devastation**						
Daily	52 (64.2%)	57 (69.5%)	0.627	1.028	0.079	109 (66.9%)
Less than daily	19 (23.5%)	14 (17.1%)				33 (20.2%)
Did not watch the TV images of the devastation	10 (12.3%)	11 (13.4%)				21 (12.9%)
**Frequency of reading newspaper/internet article related to devastation by 2020 flooding**						
Daily	62 (76.5%)	63 (77.8%)				125 (77.2%)
Less than daily	16 (19.8%)	15 (18.5%)	1.000	0.040	0.016	31 (19.1%)
Did not read newspaper and internet articles related to the devastation	3 (3.7%)	3 (3.7%)				6 (3.7%)
**Substantial home damage**						
No	84 (96.6%)	81 (93.1%)	0.496	1.055	0.078	165 (94.8%)
Yes	3 (3.4%)	6 (6.9%)				9 (0.2%)
**Lost property due to flood**						
No lost	81 (93.1%)	72 (82.8%)	0.061	4.387	0.159	153 (87.9%)
Yes lost	6 (6.9%)	15 (17.2%)				21 (12.1%)
**Support from family/friend during/after flood**						
Yes. I have had absolute support	41 (51.9%)	30 (39.0%)				71 (45.5%)
Yes. I have had some support	7 (8.9%)	15 (19.5%)				22 (14.1%)
Yes. But only limited support	3 (3.8%)	14 (18.2%)				17 (10.9%)
Not at all	28 (35.4%)	18 (23.4%)	0.003	13.882	0.298	46 (29.5%)
**Support from Red Cross during/after flood**						
Yes. I have had absolute support	7 (8.9%)	6 (7.4%)				13 (8.1%)
Yes. I have had some support	0 (0.0%)	3 (3.7%)				3 (1.9%)
Yes. But only limited support	1 (1.3%)	6 (7.4%)	0.052	8.863	0.235	7 (4.4%)
Not at all	3 (3.8%)	7 (8.6%)				10 (6.3%)
Not Applicable as I was not impacted by the floods	68 (86.1%)	59 (72.8%)				127 (79.4%)
**Support from Government of Alberta during/after flood**						
Yes. I have had absolute support	5 (6.3%)	6 (7.4%)				11 (6.9%)
Yes. I have had some support	0 (0.0%)	4 (4.9%)				4 (2.5%)
Yes. But only limited support	2 (2.5%)	4 (4.9%)				6 (3.8%)
Not at all	5 (6.3%)	7 (8.6%)	0.249	5.453	0.185	12 (7.5%)
Not Applicable as I was not impacted by the floods	67 (84.8%)	60 (74.1%)				127 (79.4%)
**Support from Insurers during/after flood**						
Yes. I have had absolute support	1 (1.3%)	4 (4.9%)				5 (3.1%)
Yes. I have had some support	1 (1.3%)	3 (3.7%)				4 (2.5%)
Yes. But only limited support	4 (5.1%)	3 (3.7%)	0.250	5.560	0.186	7 (4.4%)
Not at all	3 (3.8%)	8 (9.9%)				11 (6.9%)
Not Applicable as I was not impacted by the floods	70 (88.6%)	63 (77.8%)				133 (83.1%)

**Table 3:** Chi-square test of association between employer type and COVID-19 related variables.

Variables	Employer		*p*-Value	Chi- Square	Effect Size Phi/Cramer’s V	Overall
	School Board	Other Employment				
**Fearful contracting coronavirus**						
No	6 (7.4%)	7 (8.5%)	1.000	0.071	0.021	13 (8.0%)
Yes	75 (92.6%)	75 (91.5%)				150 (92.0%)
**Fearful for close friends/family contracting coronavirus**						
No	3 (3.7%)	3 (3.7%)	1.000	0.000	0.001	6 (3.7%)
Yes	78 (96.3%)	79 (96.3%)				157% (96.3%)
**Any close friends/family member sick from coronavirus**						
No	17 (21.0%)	27 (33.8%)	0.079	3.301	0.143	44 (27.3%)
Yes	64 (79.0%)	53 (66.3%)				117 (72.7%)
**Had to self-isolate or self-quarantine due to COVID-19 symptoms**						
No	27 (33.3%)	35 (43.2%)	0.258	1.672	0.102	62 (38.3%)
Yes	54 (66.7%)	46 (56.8%)				100 61.7%)
**Frequency of watching images of sick/dead COVID-19 people**						
Daily	39 (48.1%)	35 (42.7%)				74 (45.4%)
<Daily	32 (39.5%)	36 (43.9%)	0.802	0.493	0.055	68 (41.7%)
I did not watch the TV images of the pandemic	10 (12.3%)	11 (13.4%)				21 (12.9%)
**Frequency of reading newspaper/internet article related to pandemic**						
Daily	48 (59.3%)	50 (61.0%)				98 (60.1%)
<Daily	31 (38.3%)	31 (37.8%)	0.951	0.368	0.048	62 (38.0%0
**I did not read newspaper and internet articles related to the pandemic**	2 (2.5%)	1 (1.2%)				3 (1.8%)
Lost job due to COVID-19 pandemic						
No	73 (90.1%)	74 (90.2%)	1.000	0.001	0.002	147 (90.2%)
Yes	8 (9.9%)	8 (9.8%)				16 (9.8%)
**Support from family/friends since pandemic**						
Yes. I have had absolute support	38 (47.5%)	35 (42.7%)				73 (45.1%)
Yes. I have had some support	24 (30.0%)	28 (34.1%)	0.646	1.734	0.103	52 (32.1%)
Yes. But only limited support	9 (11.3%)	13 (15.9%)				22 (13.6%)
Not at all	9 (11.3%)	6 (7.3%)				15 (9.3%)
**Support from Government of Canada since pandemic**						
Yes. I have had absolute support	14 (17.9%)	8 (10.0%)				22 (13.9%)
Yes. I have had some support	15 (19.2%)	11 (13.8%)				26 (16.5%)
Yes. But only limited support	13 (16.7%)	11 (13.8%)				24 (15.2%)
Not at all	36 (46.2%)	50 (62.5%)	0.201	4.673	0.172	24 (15.2%)
**Support from Government of Alberta since pandemic**						
Yes. I have had absolute support	12 (15.2%)	4 (5.0%)				16 (10.1%)
Yes. I have had some support	14 (17.7%)	9 (11.3%)				23 (14.5%)
Yes. But only limited support	9 (11.4%)	10 (12.5%)	0.078	6.807	0.207	19 (11.9%)
Not at all	44 (55.7)	57 (71.3%)				101 (63.5%)
**Support from employer since pandemic**						
Yes. I have had absolute support	37 (46.3%)	39 (48.1%)				76 (47.2%)
Yes. I have had some support	29 (36.3%)	15 (18.5%)				44 (27.3%)
Yes. But only limited support	11 (13.8%)	13 (16.0%)	0.007	11.786	0.271	24 (14.9%)
Not at all	3 (3.8%)	14 (17.3%)				17 (10.6%)

**Table 4:** Chi-square test of association between employer type and mental health variables.

Variables	Employer		*p*-Value	Chi- Square	Effect Size Phi/Cramer’s V	Overall
	School Board	Other Employment				
**Have received a Mental Health Diagnosis**						
Yes	38 (43.7%)	53 (60.9%)				91 (52.3%)
No	49 (56.3%)	34 (39.1%)	0.033	5.183	0.173	83 (47.7%)
**Have been diagnosed with Depression**						
Yes	19 (21.8%)	35 (40.2%)				54 (31.0%)
No	68 (78.2%)	52 (59.8%)	0.014	6.874	0.199	120 (69.0%)
**Have been diagnosed with Anxiety**						
Yes	32 (36.8%)	42 (48.3%)				74 (42.5%)
No	55 (63.2%)	45 (51.7%)	0.167	2.351	0.116	100 (57.5)
**Currently taking antidepressants**						
Yes	26 (29.9%)	28 (32.2%)				54 (31.0%)
No	61 (70.1%)	59 (67.8%)	0.870	0.107	0.025	120 (69.0%)
**Currently taking sleeping tablets**						
Yes	8 (9.2%)	9 (10.3%)				17 (9.8%)
No	79 (90.8%)	78 (89.7%)	1.000	0.65	0.019	157 (90.2%)
**Received Mental health counselling in the past year**						
Yes	25 (28.7%)	38 (43.7%)				63 (36.2%)
No	62 (71.3%)	49 (56.3%)	0.058	4.205	0.155	111 (63.8%)
**Would like to receive mental health counselling**						
Yes	40 (46.0%)	49 (56.3%)				89 (51.1%)
No	47 (54.0%)	38 (43.7%)	0.225	1.863	0.103	85 (48.9%)
**Resilience**						
High to normal resilience	51 (65.4%)	53 (64.6%)				104 (65.0%)
Low resilience	27 (34.6%)	29 (35.4%)	1.000	0.010	0.008	56 (35.0%)
**Major Depressive Disorder (MDD)**						
MDD Unlikely	49 (64.5%)	42 (51.2%)				91 (57.6%)
MDD Likely	27 (35.5%)	40 (48.8%)	0.108	2.837	0.134	67 (42.4%)
**Generalized Anxiety Disorder (GAD)**						
GAD Unlikely	50 (66.7%)	44 (53.7%)	0.106	2.759	0.133	94 (59.9%)
GAD Likely	25 (33.3%)	38 (46.3%)				63 (40.1%)
**Post-Traumatic Stress Disorder (PTSD)**						
PTSD Unlikely	54 (72.0%)	44 (55.0%)				98 (63.2%)
PTSD Likely	21 (28.0%)	36 (45.0%)	0.031	4.811	0.176	57 (36.8%)

## Data Availability

Data associated with this study will be made freely available upon reasonable request from the corresponding author.

## Supplementary Materials

The following supporting information can be downloaded at: https://www.mdpi.com/article/10.3390/ijerph19010435/s1, Fort McMurray Trauma Study Survey Questionnaire.
