# Supplementary material for: Mental Health Impacts of Wildfire, Flooding and COVID-19 on Fort McMurray School Board Staff and Other Employees: A Comparative Study
Source: Int J Environ Res Public Health. 2021 Dec 31;19(1):435. doi: 10.3390/ijerph19010435 (PMC8744856; doi:10.3390/ijerph19010435)
Supplement: Supplementary file 1 [file ijerph-19-00435-s001.zip › ijerph-1447698-supplementary.pdf]

Baseline demographic and clinical information

1. **Gender:** Male______ Female_____
2. **Age:** < 18 ____ 18-25____ 26-40____ 41-60____ > 60____
3. **Employment:**

   Employed___

   Stay at Home Parent___

   Unemployed on social assistance_____

   Unemployed. No income_____

   Retired____

   Student__
4. **If employed, where?**

   School Boards_______

   Healthcare industry_____

   Keyano College________

   Oil Sands Industry________

   Municipal or Government Agency_______

   Other, please specify_____________________________
5. **Marital status:** Single__Married__Partnered/cohabiting___Divorced___ Widowed__
6. **Area of residence during the 2016 wildfire:**

   Abasand__

   Beacon Hill___

   Waterways/Draper ____

   Downtown___

   Timberlea___

   Thickwood___

   Wood Buffalo___

   Parsons Creek___

   Saprae Creek____

   Gregoire/Saline Creek/Prairie Creek___
7. **Area of residence during the 2020 floods:**

   Abasand__

   Beacon Hill___

   Waterways/Draper ____

   Downtown___

Timberlea___

Thickwood___

Wood Buffalo___

Parsons Creek___

Saprae Creek____

Gregoire/Saline Creek/Prairie Creek___

8. **Where did you live prior to the 2016 Fort McMurray wildfire?**

   Own home___ Renting___Hostel____Homeless___ Work camp____

9. **Where did you live prior to the 2020 Fort McMurray flooding?**

   Own home___ Renting___Hostel____Homeless___ Work camp____

10. **Where do you live now?**

    Own home___ Renting___Hostel____Homeless___Work camp____

11. **Have you received a mental health diagnosis from a health professional? Please check all that apply.**

- o Depression
- o Bipolar Disorder
- o Anxiety
- o Alcohol Abuse
- o Drug Abuse
- o Schizophrenia
- o Personality Disorder
- o I have never received a mental health diagnosis from a health professional
- o If diagnosis not listed, please specify: ______________________

12. **Are you on any of the following medication for a mental health concern? Please check all that apply.**

- o Antidepressants
- o Antipsychotics
- o Benzodiazepines
- o Mood Stabilizers
- o Sleeping Tablets
- o If medication not listed, please specify: _________________________
- o I am not on any medication for mental health concerns

13. Have you received mental health counselling in the past year?

- o Yes
- o No

14. Would you like to receive mental health counselling?

- o Yes
- o No

15. Have you used/abused any of the following drugs in the past year? (Select all that apply)

- o Downers or Sedatives (Barbiturates, etc.)
- o Benzos (Valium, Xanax, etc.)
- o Hallucinogens (including ecstasy, LSD, PCP, angle dust, mescaline, peyote, psilocybin, mushrooms)
- o Alcohol
- o Solvents/Inhalants (Thinners, gas, gasoline/petrol, glue, solution, trichlorethylene)
- o Heroin or other Opiates (Morphine, etc.)
- o Marijuana
- o GHB
- o Anabolic Steroids
- o Stimulants (cocaine, amphetamine)
- o Other (specify): ________________________________

16. Have you received addiction counselling in the past year?

- o Yes
- o No

17. Would you like to receive addiction counselling?

- o Yes
- o No

Fort McMurray 2016 Wildfire

18. Where did you live on the 3rd of May when there was an order to evacuate Fort McMurray?

In Fort McMurray__

In Alberta but not in Fort McMurray___

In another province____

19. Did you witness the burning of any homes or structures by the wildfires in Fort McMurray?

Yes___ No____

20. On the day of the evacuation, were you fearful for your life or the lives of your friends or family? Yes___ No____

21. During the period of the evacuation order for Fort McMurray, how frequently did you watch television images about the devastation caused by the wildfires in Fort McMurray?

Daily___

About every other day___

About once a week___

Less than once a week___

I did not watch the TV images of the devastation___

22. During the period of the evacuation order for Fort McMurray, how frequently did you read newspaper and internet articles related to the devastation caused by the wildfires in Fort McMurray?

Daily___

About every other day___

About once a week___

Less than once a week___

I did not watch the TV images of the devastation____

23. Did you lose property as a result of the wildfires in Fort McMurray?

Home was completely destroyed____

Home suffered substantial smoke damage____

Home suffered slight smoke damage____

Car was completely destroyed by the fire___

Business was completely destroyed by the fire___

Suffered no loss of property in the fire___

24. Do you live in the same house you lived in before the evacuation order came into effect?

Yes___

No, I live in a different house even though my previous home was **not** destroyed by the fire___

No, I live in a different house because my previous home was destroyed by the fire___

25. Did you receive sufficient support from family and friends after the evacuation order for Fort McMurray was declared?

Yes, I have had absolute support___

Yes, I have had some support___

Yes, but only limited support____

Not at all___

26. Did you receive sufficient support from the Red Cross after the evacuation order for Fort McMurray was declared?

Yes, I have had absolute support___

Yes, I have had some support___

Yes, but only limited support____

Not at all___

27. Did you receive sufficient support from the Government of Alberta after the evacuation order for Fort McMurray was declared?

Yes, I have had absolute support___

Yes, I have had some support___

Yes, but only limited support____

Not at all___

28. Did you receive sufficient support from your insurers after the evacuation order for Fort McMurray was declared?

Yes, I have had absolute support___

Yes, I have had some support___

Yes, but only limited support____

Not at all___

Not Applicable as I do not have insurers____

29. Did you receive any counselling when you returned to Fort McMurray after the wildfires?

Yes___ No ____

Fort McMurray 2020 Floods

30. Where did you live just prior to the 2020 Fort McMurray flooding?

In Fort McMurray__

In Alberta but not in Fort McMurray___

In another province____

31. Did you witness the flooding of homes or structures in Fort McMurray?

Yes___ No____

32. **Were you fearful for your life or the lives of your friends or family during the flooding?**

Yes___ No____

33. **During the 2020 Fort McMurray flooding how frequently did you watch television images about the devastation caused by the floods?**

Daily___

About every other day___

About once a week___

Less than once a week___

I did not watch the TV images of the devastation___

34. **During the 2020 Fort McMurray flooding how frequently did you read newspaper and internet articles related to the devastation caused by flooding?**

Daily___

About every other day___

About once a week___

Less than once a week___

I did not watch the TV images of the devastation____

35. **Did you lose property as a result of the floods in Fort McMurray?**

Home was completely destroyed____

Home suffered substantial damage____

Home suffered slight damage____

Car was completely destroyed by the floods___

Business was completely destroyed by the floods___

Suffered no loss of property in the floods___

36. **Do you live in the same house you lived in before the floods?**

Yes___

No, I live in a different house even though my previous home was **not** destroyed by the fire___

No, I live in a different house because my previous home was destroyed by the fire___

37. **Did you receive sufficient support from family and friends during and after the floods?**

Yes, I have had absolute support___

Yes, I have had some support___

Yes, but only limited support____

Not at all___

Not Applicable as I was not impacted by the floods____

38. Did you receive sufficient support from the Red Cross during and the floods?

Yes, I have had absolute support___

Yes, I have had some support___

Yes, but only limited support____

Not at all___

Not Applicable as I was not impacted by the floods____

39. Did you receive sufficient support from the Government of Alberta during and the floods?

Yes, I have had absolute support___

Yes, I have had some support___

Yes, but only limited support____

Not at all___

Not Applicable as I was not impacted by the floods____

40. Did you receive sufficient support from your insurers during and the floods?

Yes, I have had absolute support___

Yes, I have had some support___

Yes, but only limited support____

Not at all___

Not Applicable as I was not impacted by the floods____

COVID-19 Mental Health Impacts

41. During the pandemic, have you been fearful about contracting the corona virus?

Yes___ No____

42. Have any of your close friends or family members been sick from the corona virus disease?

Yes___ No____

43. Have you had to self-isolate or self-quarantine due to COVID-19 symptoms, recent travel, or because you were in contact with someone who may have COVID-19?

- o Yes
- o No

44. During the period of the COVID-19 pandemic, how frequently did you watch television images about of sick and dead people caused by corona virus

Daily___

About every other day___

About once a week___

Less than once a week___

I did not watch the TV images of the devastation___

45. During the period of the COVID-19 pandemic how frequently did you read newspaper and internet articles related to the pandemic

Daily___

About every other day___

About once a week___

Less than once a week___

I did not watch the TV images of the devastation____

46. Did you lose your job due to the COVID-19 pandemic??

Yes___

No__

47. Have you had sufficient support from family and friends since the COVID-19 pandemic declared?

Yes, I have had absolute support___

Yes, I have had some support___

Yes, but only limited support____

Not at all___

48. Have you had sufficient support from the Government of Canada since the COVID-19 pandemic was declared?

Yes, I have had absolute support___

Yes, I have had some support___

Yes, but only limited support____

Not at all___

49. Have you had sufficient support from the Government of Alberta since the COVID-19 pandemic was declared?

Yes, I have had absolute support___

Yes, I have had some support___

Yes, but only limited support____

Not at all___

50. Have you had sufficient support from your employer since the COVID-19 pandemic was declared?

Yes, I have had absolute support___

Yes, I have had some support___

Yes, but only limited support____

Not at all___

Not Applicable as I do not have insurers____

For each of the following statements, please indicate your level of agreement

	Strongly Disagree	Disagree	Neutral	Agree	Strongly Agree
I tend to bounce back quickly after hard times.					
I have a hard time making it through stressful events.					
It does not take me long to recover from a stressful event.					
It is hard for me to snap back when something bad happens.					
I usually come through difficult times with little trouble.					
I tend to take a long time to get over setbacks in my life.					

How often have you been bothered by the following problems over the last 2 weeks?

	**Not At All**	**Several Days**	**More Than Half the Days**	**Nearly Every Day**
Little interest or pleasure in doing things.				
Feeling down, depressed, or hopeless.				
Trouble falling or staying asleep, or sleeping too much.				
Feeling tired or having little energy.				
Poor appetite or overeating.				
Feeling bad about yourself - or that you are a failure or that you have let yourself or your family down.				
Trouble concentrating on things, such as reading the newspaper or watching television.				
Moving or speaking so slowly that other people could have noticed? Or the opposite - being so fidgety or restless that you have been moving around a lot more than usual.				
Thoughts that you would be better off dead or of hurting yourself in some way.				

How often have you been bothered by the following problems over the last 2 weeks?

	Not at All	Several Days	More Than Half the Days	Nearly Every Day
Feeling nervous, anxious, or on edge.				
Not being able to stop or control worrying.				
Worrying too much about different things.				
Trouble relaxing.				
Being so restless that it's hard to sit still.				
Becoming easily annoyed or irritable.				
Feeling afraid as if something awful might happen.				

Below are a list of problems and complaints people sometimes have in response to stressful life experiences. Please read each one carefully and choose the option that best matches how much you have been bothered by that problem in the **last month**.

	**Not at All**	**A Little Bit**	**Moderately**	**Quite A Bit**	**Extremely**
Repeated, disturbing memories, thoughts, or images of a stressful experience from the past?					
Repeated, disturbing dreams of a stressful experience from the past?					
Suddenly acting or feeling as if a stressful experience were happening again (as if you were reliving it)?					
Feeling very upset when something reminded you of a stressful experience from the past?					
Having physical reactions (e.g., heart pounding, trouble breathing, or sweating) when something reminded you of a stressful experience from the past?					
Avoid thinking about or talking about a stressful experience from the past or avoid having feelings related to it?					
Avoid activities or situations because they remind you of a stressful experience from the past?					
Trouble remembering important parts of a stressful experience from the past?					
Loss of interest in things that you used to enjoy?					
Feeling distant or cut off from other people?					
Feeling emotionally numb or being unable to have loving feelings for those close to you?					
Feeling as if your future will somehow be cut short?					
Trouble falling or staying asleep?					
Feeling irritable or having angry outbursts?					
Having difficulty concentrating?					
Being "super alert" or watchful on guard?					
Feeling jumpy or easily startled?					

[Pop up after the survey]

Thank you for completing the survey questions. If you will like to receive 12 months of free daily supportive text messages from the **FMMStrong Supportive Text Messaging Program**, please enter your phone number here_______

The **FMMStrong Supportive Text Messaging Program** is a unique evidence-based e-mental health support developed by researchers at the University of Alberta to support residents of Fort McMurray on the fifth anniversary of the 2016 wildfires. The daily messages help to address Stress, Anxiety, Depression and PTSD. You can learn about the benefits of daily supportive text messaging from this link: https://www.mdpi.com/1660-4601/18/4/2157
